# Successful Treatment of Persistent Hiccups in an Advanced Palliative Cancer Patient With Gabapentin: A Case Report

**DOI:** 10.7759/cureus.36982

**Published:** 2023-03-31

**Authors:** Sami Ayed Alshammary, Luma Abdelsalam Shraydeh Al Fraihat, Yara H Farahat, Abdulaziz Alshehri, Sami Almustanyir

**Affiliations:** 1 Department of Palliative Care, Comprehensive Cancer Center, King Fahad Medical City, Riyadh, SAU; 2 Internal Medicine, Alfaisal University College of Medicine, Riyadh, SAU

**Keywords:** palliative care, persistent hiccups, gabapentin, acute myeloid leukemia (aml), anti-epileptics

## Abstract

Hiccups may appear to be a common normal phenomenon that does not warrant treatment in the general population. However, severe and persistent hiccups can become annoying and distressing and may decrease the quality of life, especially in cancer patients. The management of hiccups remains challenging. Despite trying many pharmacological and non-pharmacological methods, there is no clear evidence to support the management guidelines. In our case, we successfully treated persistent hiccups of more than four days duration in a patient with acute myeloblastic leukemia with gabapentin.

## Introduction

In this case report, we present the case of a 70-year-old male diagnosed with acute myeloblastic leukemia (AML) who developed persistent hiccups of more than four days in duration.

Hiccups are a physiological phenomenon that results from the activation of the hiccups reflex arc involving the respiratory muscles of the chest and diaphragm modulated by peripheral phrenic, vagal, and central mid-brain [1,2]. Hiccups can be categorized according to their duration into three categories. Acute episode if the attack lasts for minutes to hours, persistent when it continues for more than 48 hours, and intractable if the hiccups last longer than a month [3]. Acute hiccups may result from drinking alcohol and hot or cold drinks in addition to gastric distention, stress, and anxiety. Severe causes of persistent hiccups include central nervous system (CNS) lesions, diaphragmatic or vagal nerve irritation, drug-induced, metabolic, surgical, infectious, psychogenic, and idiopathic [3]. Despite the cause, hiccups that are not under control lower the patient’s quality of life and mood by interfering with eating, drinking, sleeping, and social interactions. The epidemiology of hiccups in the general population has not been studied. Out of 100,000 patients visiting a general hospital, 54 were primarily diagnosed with hiccups, according to a retrospective review conducted among consecutive patients visiting a primary hospital [4]. In addition, a retrospective review of palliative care patients with advanced cancer showed that 3.5% of in-patients and 4.5% of those observed at home presented with severe chronic hiccups [5]. Many pharmacological and non-pharmacological approaches have been developed but revealed minimum evidence to support the treatment of persistent or intractable hiccups [6]. However, gabapentin may become a promising medication for treating persistent and intractable hiccups, according to observational studies done on palliative care patients with advanced cancer [7-9].

Here, we report the successful treatment of persisting hiccups with gabapentin with no adverse effects.

## Case presentation

Our patient is a 70-year-old diabetic male with depression. He was diagnosed with AML in 2018 and received induction and consolidation therapy with complete remission. The disease relapsed in July 2020, following which he was started on azacytidine/Veneto lax and achieved remission with minimal residual disease. The patient was admitted in august 2021 as a case of febrile neutropenia and sepsis. Intravenous (IV) antibiotics, vancomycin, and meropenem were administered to resolve sepsis. Two weeks following sepsis, he developed nausea and vomiting, four to five times daily of a moderate amount, green in color, mainly after oral intake, and not associated with constipation, diarrhea, or abdominal distension. There were no relieving factors. The patient was on omeprazole, escitalopram, allopurinol, and insulin aspart. On examination, he was vitally and clinically stable, and his abdominal examination was unremarkable. Lab results revealed pancytopenia and acute kidney injury (Table 1).

**Table 1:** Patient’s investigations.

Laboratory test	Patient’s results	Normal range
Urea	14 mg/dL	5–20 mg/dL
Creatinine	165 µmol/L	61.9–114.9 µmol/L
Calcium	2.4 mmol/L	2.2–2.6 mmol/L
Potassium	4.9 mmol/L	3.6–5.2 mmol/L
Hemoglobin	7.8 g/dL	14–17.5 g/dL
Platelet	18 × 10^9^/L	150–400 × 10^9^
White blood cell count	500/µL	4,500–11,000/µL

On August 26, 2021, an abdominal CT scan was done which was unremarkable. CT showed a normal caliber of the bowel loops. There were no signs of bowel obstruction or evidence of intra-abdominal infection or pathology. The patient was started on metoclopramide; however, his nausea only improved partially. Therefore, olanzapine 5 mg daily was added. After four days, nausea and vomiting had improved. During the same admission, he started to develop continuous hiccups almost all day long. On September 2, 2021, the hiccups were interrupting his sleep and were distressing to him and his family. Hiccups persisted until the palliative consultation team assessed the patient on September 6, 2021, and decided to start him on gabapentin 100 mg orally three times per day (TID). On the following day, his hiccups improved significantly, as they were reduced by 70% for one day but returned. Thus, the dose was increased to 200 mg TID, and his hiccups resolved almost completely. A follow-up appointment after two weeks showed a sustained effect with hiccups and complete resolution.

## Discussion

Hiccups are a temporary benign phenomenon found frequently in the population. Brief episodes of hiccups lasting less than 48 hours are usually not concerning, self-limiting, transient, and non-pathological. However, a systemic review of patients with advanced cancer showed that 1-9% of the patients had persistent or intractable hiccups [9]. These hiccups usually signify different pathological processes, such as infection, inflammation, and structural disorders. These pathologies can impact the nerves or their branches, including the vagus and the phrenic nerve. They can as well involve the CNS.

The pathophysiology of hiccups remains unknown, with no identifiable anatomical brain centers responsible for hiccups [10,11]. However, the central part of the hiccups reflex arc includes the C3-C5 of the spinal cord, hypothalamus, medulla, and reticular formation. They were first proposed in 1948 by Bailey [12]. The sensory component of the vagal and phrenic nerve and sympathetic fibers from T6-T12, as well as the pharyngeal branch of the glossopharyngeal nerve, carries the afferent fibers of the reflex. The efferent response is conveyed by different pathways, including the phrenic nerve, supplying the corresponding hemidiaphragm, and the external intercostal nerves of T1-T11, supplying the intercostal muscles, and the scalenus anticus nerve, supplying the scalene muscles. The recurrent laryngeal branch of the vagus nerve is responsible for glottis closure to continue the series of events in the hiccup reflex [13,14]. Neurotransmitters can affect the central part and possibly control the arc reflex. These neurotransmitters can be from central or peripheral sources. Central neurotransmitters include dopamine, serotonin, and GABA. Peripheral neurotransmitters include histamine, acetylcholine, epinephrine, and norepinephrine [15].

Hiccups may become distressful, causing fatigue, dehydration, malnutrition, insomnia, and mental stress. Studies have shown that hiccups can cause sleep deprivation, as they can persist during both non-rapid eye movement and rapid eye movement sleep [16]. Hiccups in advanced cancer patients are usually multifactorial. Elevating the symptoms is the main objective of the treatment instead of extensive investigations to establish the etiology. Many physical maneuvers have been identified as the first-line treatment of hiccups, including breath-holding spells, the Valsalva maneuver, pressing on eyeballs, and sipping cold water. If all non-pharmacological approaches have failed, then pharmacological management is warranted. Multiple drugs have been used in the literature to treat hiccups. These include metoclopramide, chlorpromazine, baclofen, nifedipine, valproic acid, anti-psychotics, gabapentin, and haloperidol. Several other trials have revealed insufficient evidence to guide the treatment plan [6].

In this case, the goal of the palliative care team was to relieve the patient’s symptoms quickly and reduce the number of investigations and side effects of medications to enhance his quality of life. Gabapentin is an anti-epileptic medication. It is typically used in the palliative care of cancer patients to control neuropathic pain. It may effectively treat hiccups by inhibiting the inspiratory muscles mediated by endogenous GABA or [17] by reducing the calcium influx to the muscles. Gabapentin can also block the voltage-gated Ca^2+^ channels at the presynaptic terminal of respiratory muscles.

Furthermore, it can supply the nucleus raphe magnus with high serotonin levels, which is the most likely cause of the GABAergic inhibitory impulse to hiccup reflex arc. In our case, we used gabapentin to resolve hiccups completely. The patient was satisfied with the management. He had no side effects and no recurrence of hiccups after two weeks of follow-up.

Gabapentin is safe, economical, and available in different formulations. Moreover, it has minimal side effects. Therefore, gabapentin can be considered in the treatment of persistent, intractable, and unmanageable hiccups in palliative care [18,19].

## Conclusions

This case report supports the previous observational studies on the successful treatment of persistent hiccups with gabapentin in an elderly population with terminal diseases as it is safe, widely available, and has no adverse reactions. Additional research is essential to guide the management of persistent and intractable hiccups to improve the quality of life in palliative care patients.
